# Extra-alveolar miniscrews: are all brands similar?

**DOI:** 10.1590/2177-6709.30.1.e2524221.oar

**Published:** 2025-03-24

**Authors:** Karina Tostes BORSATO, Raissa Mariele BERNARDINO, Lucas Arrais CAMPOS, Daniela Águida BENTO, Luiz Geraldo VAZ, Luiz Gonzaga GANDINI

**Affiliations:** 1 São Paulo State University, Dental School, Department of Pediatric Dentistry and Morphology (Araraquara/SP, Brazil).; 2 Tampere University Hospital (Tampere, Finland).; 3 Federal Institute of Education, Science and Technology of Santa Catarina (Florianópolis/SC, Brazil).

**Keywords:** Orthodontic anchorage procedures, Corrective Orthodontics, Mechanical tests, Microscopy, Electron, scanning, Procedimentos de ancoragem ortodôntica, Ortodontia corretiva, Testes mecânicos, Microscopia eletrônica de varredura

## Abstract

**Introduction:**

A wide variety of extra-alveolar orthodontic miniscrews are available.

**Objective:**

This study aims to describe and compare the design, and the topographical and mechanical properties of five distinct commercial brands of extra-alveolar miniscrews.

**Methods:**

Scanning electron microscopy (SEM), using 25×, 50×, 100×, and 200× photomicrographs, and in-vitro mechanical tests (driving torque, torsion, and pullout test) to compare extra-alveolar miniscrews (n=80, 2 mm x 12 mm) from the following brands: Bioray, DatSteel, Morelli, OBS, and Peclab.

**Results:**

Despite the 2 × 12 mm screw size, only the Morelli and Peclab miniscrews actually measured 12 mm in thread length. The studied brands revealed different thread characteristics (i.e., thread pitch and minor/major diameter). The Morelli miniscrews presented the smallest thread pitch and, consequently, the largest number of threads. Bioray, DatSteel, and OBS miniscrews presented a trapezoidal thread shape, while the others were triangular. SEM showed adequate polishing on the Morelli, OBS, and Peclab miniscrews. The insertion torque was higher than the removal torque for the DatSteel, Morelli, and OBS miniscrews during the driving torque test of the first four threads. The same result occurred with Morelli miniscrews during the driving torque test of the full screw. The resistance limits recorded in the pullout tests were significantly higher than the maximum force used in orthodontic tooth movement. The findings showed no significant differences between the results of the titanium and steel miniscrews.

**Conclusions:**

The five types of miniscrews studied presented different characteristics, so the clinician must consider the correct size, number of threads, length, polishing surface, and metallic alloy when choosing the appropriate miniscrew for an application.

## RESUMO

**Introdução:**

Existe uma grande variedade de miniparafusos extra-alveolares disponíveis.

**Objetivo:**

Este estudo descreve e compara o design, as características topográficas e propriedades mecânicas de cinco diferentes marcas comerciais de miniparafusos extra-alveolares.

**Métodos:**

Microscopia eletrônica de varredura (MEV), utilizando fotomicrografias de 25x, 50x, 100x e 200x e ensaios mecânicos *in vitro* (torque de inserção e remoção, torção e arrancamento) compararam miniparafusos extra-alveolares (n=80; 2 mm x 12 mm) das seguintes marcas: Bioray, DatSteel, Morelii, OBS e Peclab.

**Resultados:**

Somente as marcas Morelli e Peclab possuem 12mm de comprimento de roscas. Todas as marcas estudadas apresentaram designs de rosca diferentes (passo de rosca, diâmetro menor e diâmetro maior). Morelli apresentou a maior quantidade de filetes de rosca, devido ao menor passo. Bioray, Datsteel e OBS têm filete de rosca em formato trapezoidal; e as demais, em formato triangular. MEV mostrou o polimento adequado nas marcas Morelli, OBS e Peclab. O torque de inserção foi maior que o torque de remoção nas marcas DatSteel, Morelli e OBS durante a inserção e remoção das quatro primeiras roscas. O mesmo resultado foi obtido para Morelli na inserção e remoção completa do parafuso. Os limites de resistência registrados nos ensaios de arrancamento foram significativamente maiores que a força máxima utilizada na movimentação dentária ortodôntica. Não houve diferença estatisticamente significativa entre os miniparafusos constituídos de titânio e aço.

**Conclusão:**

Os miniparafusos estudados possuem características distintas; portanto, o profissional deve estar atento às suas necessidades clínicas para escolher o dispositivo, considerando o tamanho correto, número de roscas, comprimento, superfície de polimento e liga metálica.

## INTRODUCTION

Orthodontic miniscrews, or temporary anchorage devices (TADs), are widely used for maximum skeletal anchorage.^1^ ^,^ ^2^ They were developed by Gainsforth and Higley^3^ in 1945,and have continued to evolve. Considering their biomechanical versatility and minimally invasive nature, they can obtain maximum anchorage under wide or asymmetric movements in the absence of support teeth, when a technique limitation is present, or when there is a lack of patient collaboration.^4^

Extra-alveolar miniscrews are TADs placed outside the alveolar process that support the teeth roots, allowing distalization or mesialization movements without root interference. The infrazygomatic crest (IZC) and the mandibular buccal shelf (MBS) regions are considered safe and efficient areas to perform skeletal anchorage during bimaxillary distalization movements because they do not cause root interference during tooth displacement.^5^ ^-^ ^7^

Although most miniscrew failures occur at an early stage, their primary stability is paramount, and their design is a critical factor. The miniscrew design includes the screw diameter, length, thread form, pitch, thread height, presence of abutment, and screw material.^8^ ^-^ ^12^ In particular, the extra-alveolar miniscrew comprises three components: head; collar, or transmucosal portion; and active tip. The transmucosal portion must be smooth, avoiding any development of infection around the screw. In addition, employing a screw system with variable neck lengths, the clinician can select one that suits the particular implant site.^4^ The miniscrew head diameter must be wider than the transmucosal neck, to avoid potential soft tissue coverage.^13^ Furthermore, they must be made of non-toxic and biocompatible materials and present mechanical properties with favorable resistance to compression and tension. Most available screws are made of stainless steel or titanium, which have the same clinical efficiency.^14^ ^,^ ^15^

Mechanical testing is vital to ensure the orthodontic miniscrews effectiveness and safety during use. Mechanical tests performed on orthodontic miniscrews include the following: a) driving torque test, which measures the torque required to insert and remove a miniscrew from bone; b) torsion strength test, which measures the maximum torque that a miniscrew can withstand before fracture or failure; and c) pullout test, which measures the ability of a miniscrew to resist pullout forces.^16^

The evaluation of design, topographic characteristics, and mechanical properties of these devices can provide the orthodontic clinician with a better understanding of the miniscrew’s clinical performance, and develop recommendations for use. This study aims to compare, through scanning electron microscope (SEM) and mechanical tests (i.e., driving torque, torsion strength, and pullout tests), five commercial brands of extra-alveolar orthodontic miniscrews. To our knowledge, this is the first such comparison.

## MATERIAL AND METHODS

**Figure 1:**
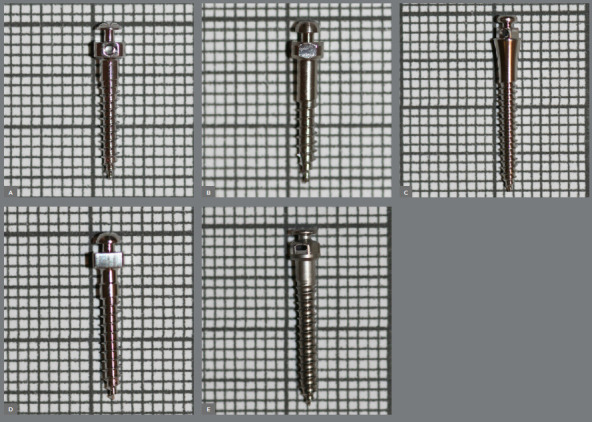
Extra alveolar miniscrews A) Bioray, B) DatSteel, C) Morelli, D) OBS, E) Peclab.

This *in-vitro* study did not require approval from the research ethics committee. Extra-alveolar miniscrews, 2 × 12 mm in size, from the following brands were analyzed: Bioray, DatSteel, Morelli, OBS, and Peclab (Fig. 1, Table 1). 

**Table 1:** Miniscrews description according to the manufacturer.

Commercial brand	Diameter (mm)	Thread length (mm)	Alloy	Insertion system	Origin country
Bioray (A)	2	12	steel	self-drilling	Taiwan
DatStell (B)	2	12	steel	self-drilling	Brazil
Morelli (C)	2	12	steel	self-drilling	Brazil
OBS (D)	2	12	steel	self-drilling	Taiwan
Peclab (E)	2	12	Ti (grade 5)	self-drilling	Brazil

The minimum sample size was calculated using the GPower program (v. 3.1.9.6 for Mac OS; Heinrich-Heine-Universität Düsseldorf, Düsseldorf, Germany; *http://www.gpower.hhu.de/*). The following parameters were considered: α = 0.05, β = 0.20, partial η^2^= 0.25, four degrees of freedom, and ten groups (interactions between two independent variables). The resulting minimum sample size was five samples per group, which was adequate to support all analyses in the study.

### TOPOGRAPHIC ANALYSIS USING SEM

To evaluate the miniscrew design and surface characteristics, one mini-screw from each brand was metallized and then mounted with double-sided tape on an aluminum base for the scanning electron microscope (JEOL IT500HR) in a high vacuum range, with 2 kV voltage acceleration. Photomicrographs of the miniscrews were obtained (25×, 50×, 100×, and 200×), showing images of each sample’s head, transmucosal portion, thread, and active tip. Interthread distances and thread depth were evaluated. In addition, a qualitative chemical analysis of the miniscrews was conducted using X-ray dispersive energy (EDS) with a voltage acceleration of 10kV.^17^

The ImageJ software was used to measure the interthread distance, thread depth, external thread diameter, internal thread diameter, and transmucosal length of each miniscrew, using the 50× SEM images. The total length of the thread was measured with a digital caliper. In the present article, the SEM images are described, but a statistical analysis is not present.

### MECHANICAL TESTS

For the driving torque tests, a driving torque tester was used (CME 30 Nm; Oswaldo Frisola, Brazil), according to the American Society for Testing and Materials (ASTM) standard F543:2017, evaluating five miniscrews of each brand in each test.

### DRIVING TORQUE TESTS

The experimental artificial bone block (4.5 × 9.5 × 3.1 cm with a 2 mm 15PCF-CP2 cortical layer; Nacional Ossos, Brazil) was fitted to the test machine. Each sample was inserted perpendicular to the test block without a pilot hole, maintaining approximately 16mm between the insertions. Using the insertion screwdriver of each model, a uniform speed of 5 rpm was applied and obtained a torque (Nm) versus angle (degrees) curve for each miniscrew tested, using the software DinaView Torque Standard/Pro M. The insertion torque was the maximum reading recorded during the four initial revolutions of the sample and at complete insertion. The removal torque was measured by reversing the direction of rotation and recording the maximum torque while removing the screw from the test block. An axial load of 1.14 kgf was used to maintain the tip of the screwdriver in the screw head during the insertion and removal procedures.^16^

### MAXIMUM TORQUE (FRACTURE TORQUE) AND RUPTURE ANGLE

For the maximum torque test, five miniscrews of each brand were assessed. The screw body was fixed in a pressure clamp to prevent rotation. The test was conducted at a constant speed of 1 rpm, applying torsion with a constant axial load of 12 N, obtaining a torque versus angle curve for each miniscrew. The screwdriver (bit) had a diameter of 5.00 mm and an exposed length of approximately 16 mm. Each specimen was submitted to the torsion test until its rupture and then visually analyzed with the aid of SEM in increments of 25×, 50×, 100×, and 200×.

### PULLOUT TEST

Finally, the pullout tests were performed according to ASTM F543:2017 using EMIC equipment (DL10000; EMIC, Brazil). Each sample (five miniscrews of each brand) was inserted in a bone block (4.5 × 9.5 × 3.1 cm with a 2 mm 15PCF-CP2 cortical layer; Nacional Ossos, Brazil), with a mean distance of 19.2 mm between each specimen and an insertion speed of 3 rpm until reaching a depth of 60% of the screw thread length. The test block and the block clamp were fixed to the base of the testing frame, so that the longitudinal axis of the screw was aligned with the direction of the applied load. A tensile loading speed was applied to the specimen at 5 mm/min until the screw broke or loosened from the test block. Force-displacement data were recorded, and the peak load at failure (limit force) was obtained from the data file. The failure mode was recorded for each (e.g., screw shaft, screw threads, or material failure).^16^

### DATA ANALYSIS

The assumption of normality was initially assessed using the Shapiro-Wilk test. For the data presenting a non-severe violation of the normal distribution (Shapiro-Wilk test p > 0.05), the assumption of homoscedasticity was evaluated using Levene’s test. No homoscedasticity (Levene’s test p < 0.05) was observed between the groups for the maximum insertion and removal torque variables. Thus, a non-parametric two-way analysis of variance (ANOVA) was employed, followed by a *post-hoc* LSD (least significant difference) test, to compare the groups for these variables. “Brand” and the “quantity of inserted threads” (i.e., four threads or all threads) were the independent variables (factors) in the analysis.

To address the objectives of the torque (breakage) and pullout tests for the data that did not present a close approximation to a normal distribution (Shapiro-Wilk test, p < 0.05), a comparison using the Kruskal-Wallis test was performed, followed by Dunn’s *post-hoc* test. For the data presenting a non-severe violation of the normal distribution (Shapiro-Wilk test, p > 0.05) and homoscedasticity (Levene’s test, p > 0.05), a one-way ANOVA was used, followed by Tukey’s *post-hoc* test. If heteroscedasticity was observed (Levene’s test p < 0.05), an ANOVA with Welch’s correction followed by the Games-Howell *post-hoc* test was used. A significance level of 5% was adopted for decision-making. Data analyses were performed using IBM SPSS Statistics 28 software (IBM Corp., Armonk, N.Y., USA). 

## RESULTS

**Figure 2:**
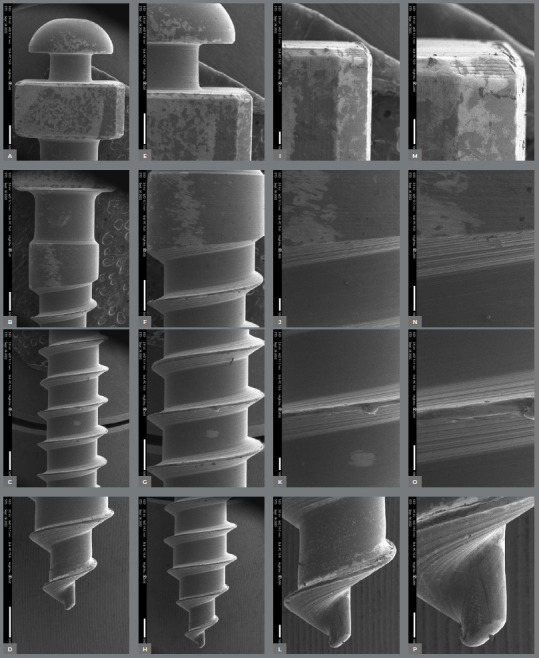
Bioray miniscrews photomicrographs with 25× **(A-**D), 50× **(E-**H), 100× **(I-**L), and 200× **(M-**P) magnification; head, transmucosal, thread, and active tip images.

The surface characteristics of the miniscrews analyzed using SEM are presented in Table 2. Figures 2 to [Fig f3] [Fig f4] [Fig f5] 6 present photomicrographs with 25×, 50×, 100×, and 200× magnification, respectively, of the head, transmucosal area, and thread of the studied miniscrews samples.

**Table 2:** Miniscrews features.

Commercial brand	Number of threads	Inter-thread distance (mm)	Thread depth (mm)*	Thread total length (mm)	Transmucous length (mm)	Head length (mm)	External thread diameter (mm)*	Internal thread diameter (mm)*	1^th^ to 4^th^ thread distance (mm)	Average speed (m/s)
Bioray (A)	13	0.85	0.27	9.5	2	3.5	1.94	1.39	34	7.08 x 10^-4^
DatSteel (B)	9	0.85	0.25	8	4	3.35	2.02	1.50	32	6.66 x 10^-4^
Morelli (C)	20	0.62	0.24	12	4.5	2.50	2.07	1.46	28	5.83 x 10^-4^
OBS (D)	11	0.75	0.30	9.5	2	3.5	1.93	1.29	32	6.66 x 10^-4^
Peclab (E)	16	0.75	0.25	12	2	3.25	2.05	1.55	32	6.66 x 10^-4^

*Measurements performed on the average thread of the screws.

**Figure 3: f3:**
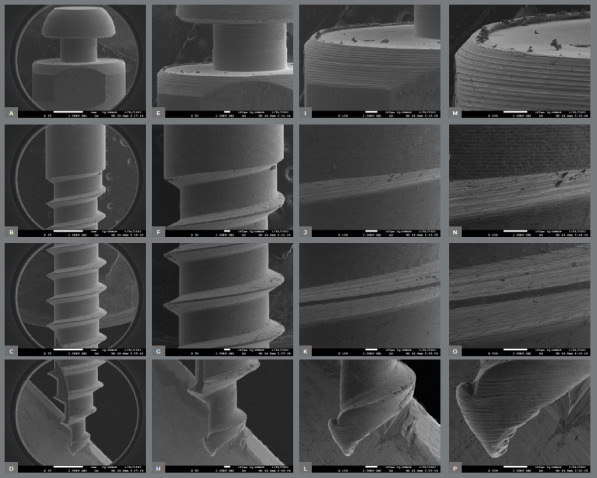
DatSteel miniscrews photomicrographs with 25× **(A-**D), 50×**(E-**H), 100× (**I-**L), and 200× **(M-**P) magnification; head, transmucosal, thread, and active tip images.

**Figure 4: f4:**
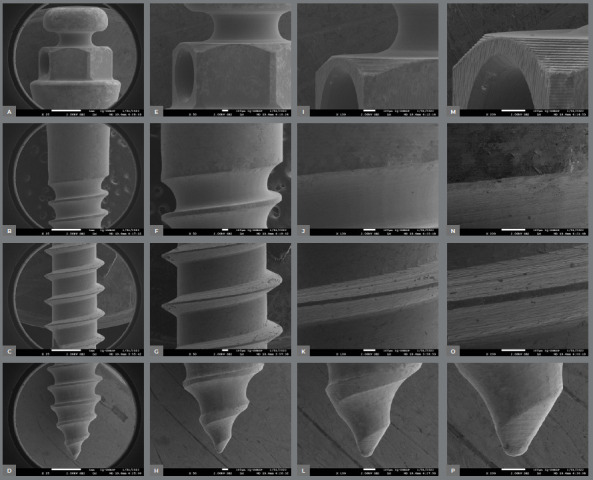
Morelli miniscrews photomicrographs with 25x **(A-**D), 50x,**(E-**H) 100x **(I-**L), and 200× **(M-**P) magnification; head, transmucosal, thread, and active tip images.

**Figure 5: f5:**
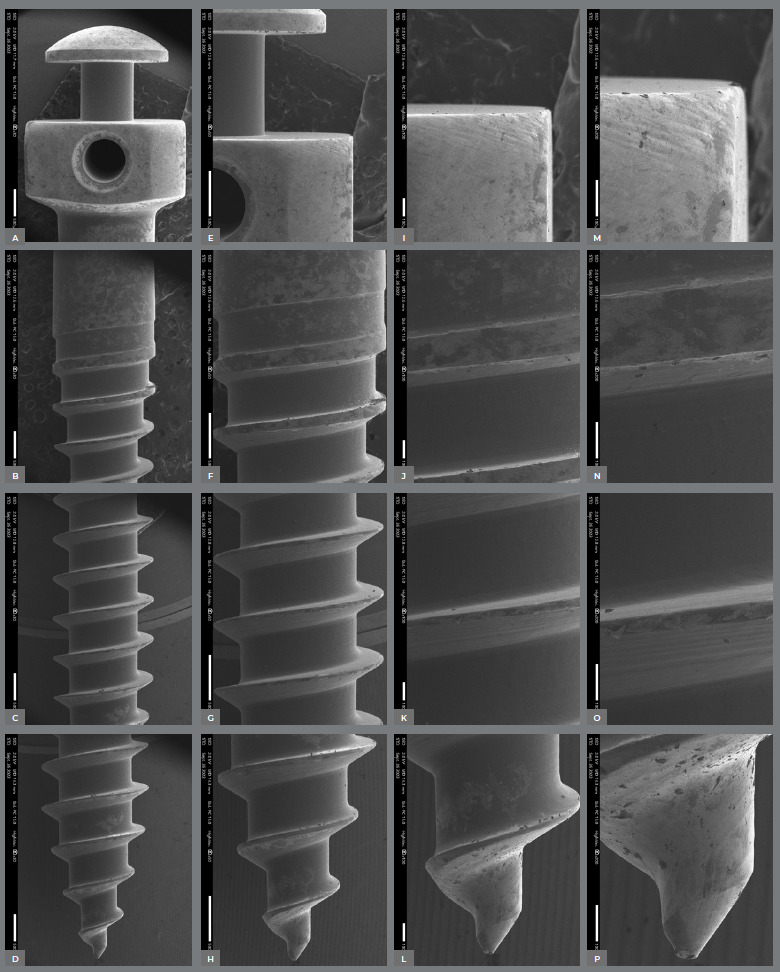
OBS miniscrews photomicrographs with 25x **(A-**D), 50x**(E-**H), 100x **(I-**L), and 200× **(M-**P) magnification; head, transmucosal, thread, and active tip images.

**Figure 6:**
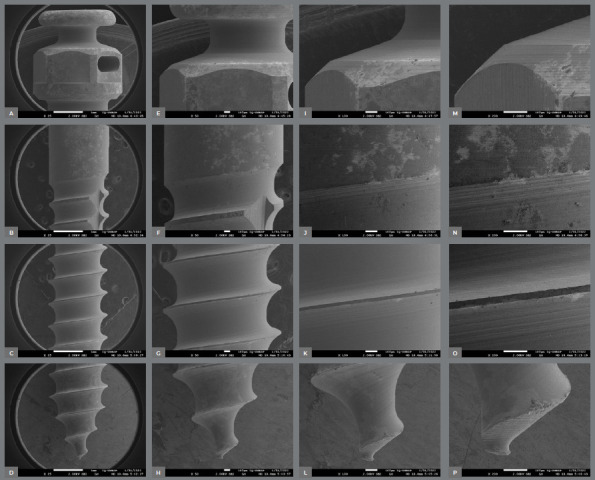
Peclab miniscrews photomicrographs with 25x **(A-**D), 50x**(E-**H), 100x **(I-**L) , and 200× **(M-**P) magnification; head, transmucosal, thread, and active tip images.

**Figure 7:**
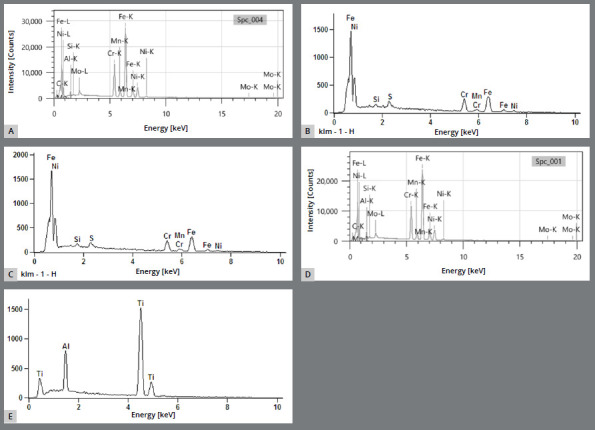
Graphs representing the semi-quantitative chemical analysis of the miniscrews: **A)** Bioray, **B)** DatSteel, **C)** Morelli, **D)** OBS, **E)** Peclab).

Figures 7A-E represent the semi-quantitative chemical analysis of the steel and titanium miniscrews using EDS with a voltage acceleration of 10 kV.

Table 3 describes the results of the partial (first four threads) and total driving torque tests, the maximum torque test, and the pullout test of all analyzed samples.

**Table 3:** Descriptive values of all tests performed.

Commercial brand	Driving torque test (Insertion and removal torque tests) full miniscrew						Driving torque test (Insertion and removal torque tests) 4 first threads						Maximum torque test (Torsion test)						Pullout test						
	Maximum insertion torque (Nm)			Maximum removal torque (Nm)			Maximum insertion torque (Nm)			Maximum removal torque (Nm)			Maximum flow torque (Nm)			Maximum torque (Nm)			Angle (degrees)			Rupture place (ST)	Resistance strength limit		
	Mean	Standard deviation	Uncertainty (U)	Mean	Standard deviation	Uncertainty (U)	Mean	Standard deviation	Uncertainty (U)	Mean	Standard deviation	Uncertainty (U)	Mean	Standard deviation	Uncertainty (U)	Mean	Standard deviation	Uncertainty (U)	Mean	Standard deviation	Uncertainty (U)		Mean	Standard deviation	Uncertainty (U)
Bioray	0.229	0.0083	0.0079	0.431	0.0086	0.0081	0.0994	0.0087	0.0079	0.16	0.017	0.015	0.504	0.031	0.028	0.579	0.024	0.022	120	17	15	4º and 5º	548	38	48
DatSteel	0.1682	0.0043	0.0042	0.2517	0.0067	0.0062	0.1416	0.0084	0.0077	0.108	0.013	0.012	0.821	0.019	0.017	0.847	0.023	0.021	80	5.5	4.9	1º	299	12	36
Morelli	0.344	0.018	0.016	0.337	0.036	0.033	0.0947	0.0105	0.0095	0.067	0.012	0.011	0.6532	0.0103	0.0093	0.7254	0.0096	0.088	100	4.5	4	5º	668	11	35
OBS	0.2227	0.0094	0.0086	0.417	0.021	0.019	0.1186	0.0051	0.0048	0.1974	0.0105	0.0096	0.543	0.034	0.031	0.653	0.02	0.018	100	5.5	4.9	1º, 2º and 3º	476	27	42
Peclab	0.302	0.0024	0.022	0.248	0.025	0.023	0.1438	0.0075	0.0069	0.119	0.04	0.036	0.604	0.018	0.016	0.75	0.06	0.05	180	5.5	4.9	5º	575	55	60

* ST: screw thread.

**Figure 8:**
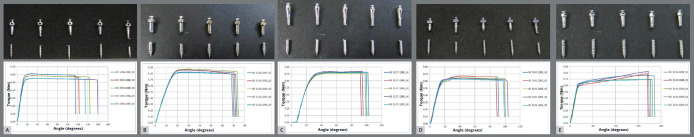
Specimens after the maximum torque test and their respective curves Torque (Nm) X Angle (degrees): **A)** Bioray, **B)** DatSteel, **C)** Morelli, **D)** OBS, **E)** Peclab.

Figure 8 shows the screws after the torsion test. All the screws fractured in the thread portion after the test; however, at different heights, but all above the middle region of the thread.

**Figure 9:**
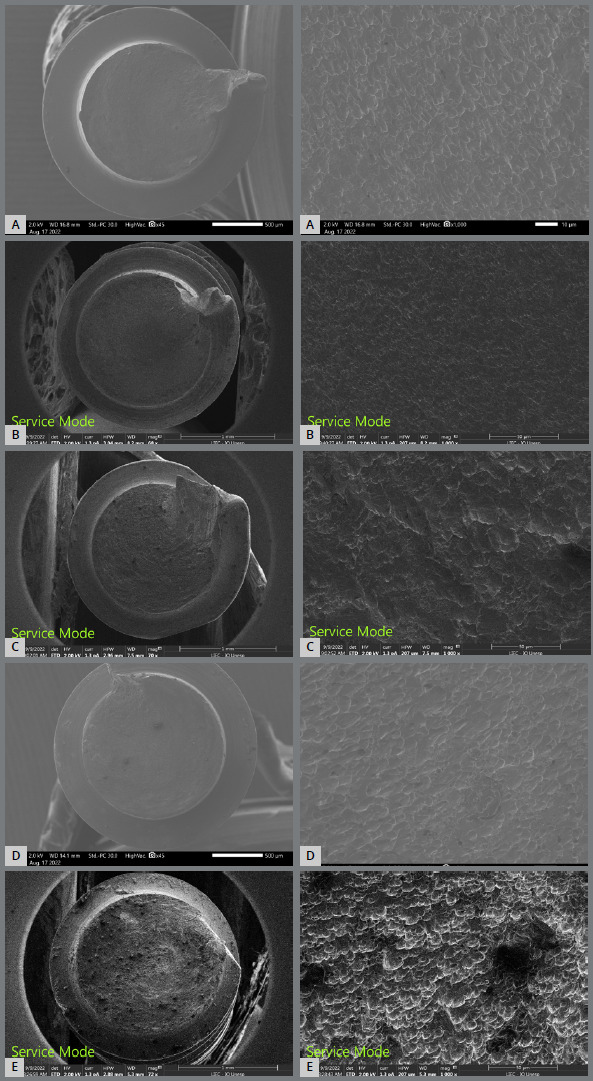
Miniscrew segments SEM after the maximum torque test, in 45x and 1000x: **A)** Bioray, **B)**DatSteel, **C)** Morelli, **D)** OBS, **E)**Peclab).

Figure 9 presents the screws SEM after the maximum torque test. In the 45× image, the surface of the spiral screw and lines of deformation have spread through the thread; at 1000×, the typical elongated undulations can be seen (shear dimples). 

**Figure 10:**
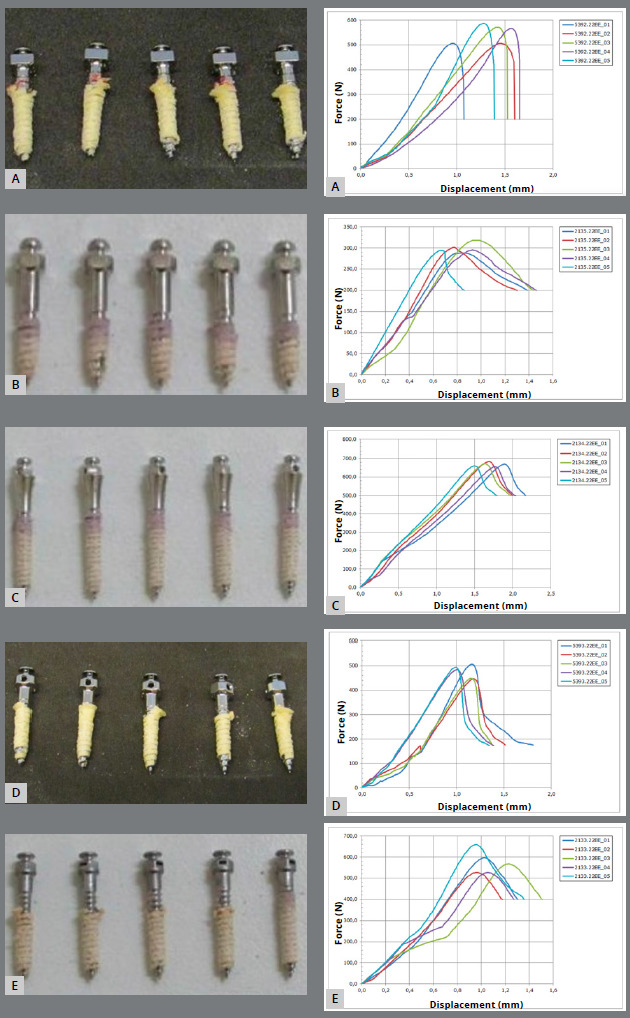
Miniscrews after the static pullout test, and their respective Force (N) x Displacement (mm) curves: **A)** Bioray, **B)**DatSteel, **C)**Morelli, **D)** OBS, **E)**Peclab).

Miniscrews images after the static pullout test are shown in Figure 10, in which Figure 10B (DatSteel sample) shows the lowest values.

Table 4 compares the driving torque test median results among the groups, according to the miniscrew brand and number of inserted threads. The results show significant differences between the maximum insertion and removal torque.

**Table 4:** Median ( interquartile range ) and the maximum insertion and removal comparison torque ( Ncm ) according to the trademark and threads inserted

	Maximum insertion torque		Maximum removal torque	
Commercial brand	First four threads	Full screw	First four threads	Full screw
Bioray (A)	10 (1.5)^Aa^	22.8 (1.5)^Bb^	15.7 (2.6)^Ac^	42.9 (1.2)^Bc^
DatSteel (B)	14.3 (1.5)^Ac^	16.7 (0.8)^Ba^	10.4 (2.3)^Ab^	25.2 (1.2)^Ba^
Morelli (C)	9.5 (1.7)^Aa^	34.4 (4.8)^Bd^	6.6 (2.5)^Aa^	33.7 (7.0)^Bb^
OBS (D)	11.8 (1.0)^Ab^	22.4 (1.8)^Bb^	19.5 (2.0)^Ad^	41.9 (3.7)^Bc^
Peclab (E)	14.5 (1.1)^Ac^	30.8 (4.2)^Bc^	11.5 (6.3)^Ab^	24.0 (5.0)^Ba^
Test statistics^#^	F_group*screw_=62.1		F_group*screw_= 13.5	
p	<0.001		<0.001	
Effect size	0.861		0.574	

# F: Non-parametric two-way ANOVA followed by LSD *post-hoc* test and epsilon squared as effect size. Different superscript lowercase letters indicate statistical difference between the groups, considering the same number of threads and response variable (comparison between rows within the same column), different superscript uppercase letters indicate statistical difference between the number of threads, considering the same brand and response variable (comparison between columns within the same row), α=5%.

Table 5 compares the mean results of the maximum torque and pullout test, according to brand. Some brands showed significant differences in the breaking angle, yield torque, and breaking torque.

**Table 5:** Measures of central tendency ( mean or median ) and variability ( standard deviation or interquartile range ), along with comparison results from torsion and pullout tests.

	Torsion test		Pullout test	
Commercial brand	Yield torque (Ncm)^1^	Breaking torque (Ncm)^1^	Breaking angle (degrees)^2^	Pullout strength (N)^1^
Bioray (A)	50.4 (3.1)^a^	57.9 (2.4)^a^	130 (30)^bc^	547.6 (38.7)^b^
DatSteel (B)	82.1 (1.9)^d^	84.7 (2.3)^d^	80 (10)^a^	299.2 (11.5)^a^
Morelli (C)	65.3 (1.0)^c^	72.5 (1.0)^c^	100 (5)^ABC^	668.0 (10.9)^c^
OBS (D)	54.3 (3.4)^a^	65.2 (2.0)^b^	100 (10)^ab^	475.6 (27.3)^b^
Peclab (E)	60.4 (1.8)^b^	75.2 (6.2)^c^	180 (10)^c^	575.0 (55.1)^BC^
Test statistics^#^	F = 131.0	FW = 77.5	H = 22.8	FW = 563.2
p	<0.001	<0.001	<0.001	<0.001
Effect size	0.963	0.904	0.942	0.945

^1^Values are presented as mean (standard deviation). ^2^Values are presented as median (interquartile range).

# F: ANOVA followed by Tukey’s *post-hoc* test and partial eta squared as effect size. FW: Welch’s ANOVA followed by Games-Howell *post-hoc* test and partial eta squared as effect size. H: Kruskal-Wallis followed by Dunn’s *post-hoc* test and epsilon squared as effect size. Different superscript lowercase letters indicate statistical difference between the brands according to the response variable (comparison between rows within the same column), α=5%.

## DISCUSSION

Accurate and careful evaluation and comparison of orthodontic miniscrews characteristics is of great value for the clinical application of these materials.

The information presented by the manufacturers regarding the miniscrew dimensions is essential in choosing an appropriate TAD for an specific installation site.^4^ According to the manufacturers, all the studied miniscrews should have a 2-mm diameter and 12-mm length in the thread portion. However, the measured diameters ranged between 1.93 and 2.07 mm. The 12-mm thread length was found in the Morelli and Peclab brands, while the other commercial brands revealed smaller measurements (Table 2). Despite this difference, the literature showed that the miniscrew insertion depth was more critical than its location or length, with a recommended depth of at least 6 mm for an effective orthodontic miniscrew.^18^ All brands have more than 6 mm in the thread portion.

The number of threads and the distance between them are also relevant characteristics for orthodontic miniscrew installation. In this study, it was observed that all brands have distinct quantities of threads and thread pitches. Morelli miniscrew showed the shortest thread pitch and, consequently, more threads; and inverse characteristics were found in the DatSteel miniscrews. The maximum effective stress decreased gradually as the screw pitch decreased, and a shorter distance between threads yielded a lower concentration of stresses.^10^ Using the insertion test for the first four threads and the length of this portion, the average speed of this movement was calculated (Table 2 ) and found a lower average speed with a smaller thread pitch. Clinically, extra-alveolar miniscrews with tight threads are installed at a lower average speed than those with threads with greater pitch. 

The miniscrews should present a smooth surface, with adequate polishing, to minimize biofilm accumulation on the exposed surface and peri-implant tissue.^4^ The 200× SEM of the miniscrew head showed the proper polishing in the Morelli, OBS, and Peclab brands. The other brands showed burrs on the surfaces, which increase the possibility of biofilm accumulation, inflammation of surrounding periodontal tissue, and, consequently, miniscrew failure (Fig. 2-6).

The smooth and polished surface of orthodontic miniscrews may justify the lack of osseointegration, although self-drilling screws have greater bone contact than conventional implants.^19^ Thus, the primary stability of the miniscrew is influenced by the bone contact during insertion,^20^ and the applied load to the screw head should not be greater than the resistance force limit.^16^ The torsion to rupture test verified significant differences between the studied brands (Table 5). Even with this difference, the maximum insertion torque did not exceed the miniscrews yield strength. It is important to emphasize that this result was obtained in a laboratory, and should not be transported directly to the clinic. The obtained torque values are dangerously high for application in the mouth. The ideal installation torque is 10-15 Ncm for long-term stability.

All studied miniscrews have a conical shape and self-drilling capability. The active tip of the mini-screw must be extremely fine and sharp to rule out the need for prior drilling.^4^ This characteristic was evidenced in the five commercial brands. In addition, all studied brands have a mushroom-shaped head with a hole for the force application (Fig. 2-6). The thread design is also different between the evaluated brands. Bioray, DatSteel, and OBS miniscrews have a trapezoidal thread shape, while the others are triangular (Fig. 2-6). This characteristic can influence the risk of miniscrew failure. According to Chang et al.,^21^ the trapezoidal threads engaged more bone than the triangular threads, and the increased core diameter in the tapered portion also helped to decrease the chance of breakage or bending failure in this high-stress area.

The semi-quantitative analysis graphs (Fig. 7) show satisfactory iron, nickel, silicon, selenium, and chromium levels in the stainless steel commercial brands (Bioray, DatSteel, Morelli, and OBS). Titanium and aluminum are also at appropriate levels in the composition of the titanium grade 5 alloy miniscrews (Peclab)^22^, and did not influence the maximum insertion and removal torques. No statistically significant difference was present between the groups. 

Additionally, the material composition was not an influencing factor in the maximum resistance; the averages presented in Table 4 show the steel miniscrews with greater and lesser resistances when compared to the titanium miniscrews. Thus, it was verified that these titanium alloy screws present less variability when compared to the steel screws. The literature presents the clinical efficacy of stainless steel and titanium in the constitution of the orthodontic miniscrews.^14^ ^,^ ^15^

The insertion torque in the first four threads was statistically lower than that of all-thread insertion due to the smaller diameter at the four initial threads of the conical miniscrews. With the increase in thread diameter, an increase in torque is evident. Thus, there is a greater risk of fracture at the end of the installation. After the torsion test, all screws were fractured in the thread portion; however, at different heights, but all above the thread’s middle portion. According to Lee et al.^13^, the fractures in this region are the result of the material’s intrinsic limiting factor. The same ductile fracture behavior and typical elongated undulations (shear dimples)^22^ were observed in all samples, with no significant differences based on the alloy composition (Fig. 9).

The insertion torque should be higher than the removal torque in *in-vitro* tests.^23^ This present results corroborate with the literature in the results obtained from the commercial brands B (DatSteel), C (Morelli), and E (Peclab) during the insertion and removal of the first four threads; and from C and E with the complete screw insertion and removal. Brands A (Bioray) and D (OBS) during the four-thread torque test and A, B, and D in the total insertion of the threads showed higher torque during removal. This behavior is probably due to the thread design, which presents an angulation that provides resistance to the counter-torque, that is, an anti-rotation, important in movements that “unscrew” the miniscrew during activation.^17^ Despite having the insertion torque lower than the removal torque, brand B does not have an angled thread, as explained by the fewer threads and lower depth (Table 2). Clinically, during the insertion process at IZC and MBS areas, an inclination of the miniscrews (approximately 60°) is required, reaching more cortical bone, so the insertion torque would likely be higher.^5^ ^,^ ^24^ ^-^ ^26^

A study performed with extra-alveolar screws in dogs obtained an average pullout resistance of 388.3 ± 23.1 N in the mandible posterior region.^20^ The values obtained in the present study were higher, except for brand B (299 N). The force required for orthodontic tooth movement ranges from 0.3 to 4 N,^27^ much lower than the resistance limits recorded in the traction tests (Table 5). Of note, brand B, whose strength in the resistance limit was the lowest, has the fewest threads and the largest pitch. Thus, this design is more susceptible to pullout than the others.

The information obtained in this study is relevant, as it provides safety for the clinician using miniscrews, mainly related to the force applied during installation. Furthermore, the present results showed safety for both steel and titanium extra-alveolar miniscrews.^23^ However, the clinician must pay attention when choosing the device size, checking whether the size provided by the manufacturer refers to the total screw length, only the thread part, or the thread portion plus the neck of the screw, avoiding possible complications, such as maxillary sinus perforation. 

The limitations of this study refer to the impossibility of accurately replicating all clinical conditions; however, care was taken in choosing the appropriate methodology. Future clinical studies should be carried out to confirm the present results.

## CONCLUSION

After describing and comparing the design, topographic characteristics, and mechanical properties of five distinct brands of extra-alveolar orthodontic miniscrews commercial brands, this study concludes the following:

» The 12-mm thread length was found in the Morelli and Peclab brands. The number of threads differs among the commercial brands studied. Morelli presented the shortest screw pitch.
» Miniscrew surface polishing was adequate for Morelli, OBS and Peclab brands.
» The torsion test until rupture verified significant differences between the studied brands. The maximum insertion torque did not exceed the miniscrews breaking torque.
» The titanium and steel alloys behaved similarly in the maximum insertion and removal tests. However, the titanium miniscrews presented less variability, when compared to the steel miniscrews, in the maximum resistance to the flow in the torsion test.
» Although a significant difference in tensile strength was observed, the values obtained were higher than the mean force used in orthodontic tooth movement.

## References

[1] Almeida MR (2019). Biomechanics of extra-alveolar mini-implants. Dental Press J Orthod.

[2] Chen G, Teng F, Xu TM (2016). Distalization of the maxillary and mandibular dentitions with miniscrew anchorage in a patient with moderate Class I bimaxillary dentoalveolar protrusion. Am J Orthod Dentofacial Orthop.

[3] Gainsforth BL, Higley L (1945). A study of orthodontic anchorage possibilities in basal bone. Am J Orthod Oral Surg.

[4] Melsen B (2005). Mini-implants Where are we?. J Clin Orthod.

[5] Chang CH, Lin JS, Roberts WE (2019). Failure rates for stainless steel versus titanium alloy infrazygomatic crest bone screws a single-center, randomized double-blind clinical trial. Angle Orthod.

[6] Chang C, Liu SS, Roberts WE (2015). Primary failure rate for 1680 extra-alveolar mandibular buccal shelf mini-screws placed in movable mucosa or attached gingiva. Angle Orthod.

[7] Hsu E, Lin J, Yeh H, Chang C, Roberts E (2017). Comparison of the failure rate for infra- zygomatic bone screws placed in movable mucosa or attached gingiva. Int J Orthod Implant.

[8] Kim YK, Kim YJ, Yun PY, Kim JW (2009). Effects of the taper shape, dual-thread, and length on the mechanical properties of mini-implants. Angle Orthod.

[9] Lim SA, Cha JY, Hwang CJ (2008). Insertion torque of orthodontic miniscrews according to changes in shape, diameter and length. Angle Orthod.

[10] Motoyoshi M, Yano S, Tsuruoka T, Shimizu N (2005). Biomechanical effect of abutment on stability of orthodontic mini-implant A finite element analysis. Clin Oral Implants Res.

[11] Song YY, Cha JY, Hwang CJ (2007). Mechanical characteristics of various orthodontic mini-screws in relation to artificial cortical bone thickness. Angle Orthod.

[12] Wilmes B, Ottenstreuer S, Su YY, Drescher D (2008). Impact of implant design on primary stability of orthodontic mini-implants. J Orofac Orthop.

[13] Lee JS, Kin JK, Park YC, Vanarsdall RL (2007). Application of orthodontic mini-implants.

[14] Mecenas P, Espinosa DG, Cardoso PC, Normando D (2020). Stainless steel or titanium mini-implants. Angle Orthod.

[15] Brown RN, Sexton BE, Gabriel Chu TM, Katona TR, Stewart KT, Kyung HM (2014). Comparison of stainless steel and titanium alloy orthodontic miniscrew implants a mechanical and histologic analysis. Am J Orthod Dentofacial Orthop.

[16] ASTM International (2017). ASTM. F543-17: standard specification and test methods for metallic medical bone screws.

[17] Squeff LR, Bernard M, Simonson DA, Elias CN, Nojima LI (2008). Caracterização de mini-implantes utilizados na ancoragem ortodôntica. Rev Dent Press Ortodon Ortop Facial.

[18] Tseng YC, Hsieh CH, Chen CH, Shen YS, Huang IY, Chen CM (2006). The application of mini-implants for orthodontic anchorage. Int J Oral Maxillofac Surg.

[19] Prager T, Holtgrave E (2003). Primary stability of self-drilling and conventional screw implants for orthodontic anchorage. J Dent Res.

[20] Huja SS, Litsky AS, Beck FM, Johnson KA, Larsen PE (2005). Pull-out strength of monocortical screws placed in the maxillae and mandibles of dogs. Am J Orthod Dentofacial Orthop.

[21] Chang JZ, Chen YJ, Tung YY, Chiang YY, Lai EH, Chen WP (2012). Effects of thread depth, taper shape, and taper length on the mechanical properties of mini-implants. Am J Orthod Dentofacial Orthop.

[22] Koterazawa Y (1974). Fractography. J Soc Mater Sci.

[23] Suzuki EY, Suzuki B (2011). Placement and removal torque values of orthodontic miniscrew implants. Am J Orthod Dentofac Orthop.

[24] Liu H, Wu X, Tan J, Li X (2019). Safe regions of miniscrew implantation for distalization of mandibular dentition with CBCT. Prog Orthod.

[25] Sreenivasagan S, Subramanian AK, Nivethigaa B (2021). Assessment of insertion torque of mini-implant and its correlation with primary stability and pain levels in orthodontic patients. J Contemp Dent Pract.

[26] Nucera R, Lo Giudice A, Bellocchio AM, Spinuzza P, Caprioglio A, Perillo L (2017). Bone and cortical bone thickness of mandibular buccal shelf for mini-screw insertion in adults. Angle Orthod.

[27] Ren Y, Maltha JC, Kuijpers-Jagtman AM (2003). Optimum force magnitude for orthodontic tooth movement a systematic literature review. Angle Orthod.

